# Validity and Reliability of the Brazilian Version of the Rapid Estimate of Adult Literacy in Dentistry – BREALD-30

**DOI:** 10.1371/journal.pone.0131600

**Published:** 2015-07-09

**Authors:** Monica C. Junkes, Fabian C. Fraiz, Fernanda Sardenberg, Jessica Y. Lee, Saul M. Paiva, Fernanda M. Ferreira

**Affiliations:** 1 Department of Stomatology, Universidade Federal do Paraná, Curitiba, Paraná, Brazil; 2 Department of Pediatric Dentistry, University of North Caroline, Chapel Hill, North Carolina, United States of America; 3 Department of Pediatric Dentistry and Orthodontics, Universidade Federal de Minas Gerais, Belo Horizonte, Minas Gerais, Brazil; University of North Carolina at Chapel Hill, UNITED STATES

## Abstract

**Objective:**

The aim of the present study was to translate, perform the cross-cultural adaptation of the Rapid Estimate of Adult Literacy in Dentistry to Brazilian-Portuguese language and test the reliability and validity of this version.

**Methods:**

After translation and cross-cultural adaptation, interviews were conducted with 258 parents/caregivers of children in treatment at the pediatric dentistry clinics and health units in Curitiba, Brazil. To test the instrument's validity, the scores of Brazilian Rapid Estimate of Adult Literacy in Dentistry (BREALD-30) were compared based on occupation, monthly household income, educational attainment, general literacy, use of dental services and three dental outcomes.

**Results:**

The BREALD-30 demonstrated good internal reliability. Cronbach’s alpha ranged from 0.88 to 0.89 when words were deleted individually. The analysis of test-retest reliability revealed excellent reproducibility (intraclass correlation coefficient = 0.983 and Kappa coefficient ranging from moderate to nearly perfect). In the bivariate analysis, BREALD-30 scores were significantly correlated with the level of general literacy (*r_s_* = 0.593) and income (*r_s_* = 0.327) and significantly associated with occupation, educational attainment, use of dental services, self-rated oral health and the respondent’s perception regarding his/her child's oral health. However, only the association between the BREALD-30 score and the respondent’s perception regarding his/her child's oral health remained significant in the multivariate analysis.

**Conclusion:**

The BREALD-30 demonstrated satisfactory psychometric properties and is therefore applicable to adults in Brazil.

## Introduction

The ability of an individual to obtain and comprehend basic health information to make appropriate decisions is defined as health literacy [1]. Individuals with a low degree of health literacy may exhibit unhealthy behaviors and a low level of health knowledge as well as use preventive healthcare services less, leading to a greater number of hospitalizations and higher healthcare costs [2–6]. A systematic review [7] also found significant associations between low health literacy, poorer health outcomes and poorer use of health care services.

Although several studies on health literacy are found in the literature, investigations addressing dental health literacy remain scarce. The Working Group on Functional Health Literacy of the National Institute of Dental and Craniofacial Research (NID-CR)[8] stated that progress in dental studies on literacy and its effects on dental health would be difficult without instruments for assessing people's dental health literacy. Indeed, until 2007, no assessment tool was available for the determination of literacy in dentistry [9]. An individual's ability to understand and use information may vary according to his/her familiarity with the context and the vocabulary being used [10], or depending upon the medical condition being treated [11]. Thus, a study that measure health literacy using a disease-specific instrument will have greater power to detect associations with health outcomes than a study that uses a general measure of health literacy [11]. Since functional literacy seems to be context specific [10], the correlation between dental and medical literacy may not be strong enough to support the use of one of the existing medical literacy instruments in researches involving dental health literacy [12].Believing that dental health literacy may be distinct from medical health literacy and could have an independent effect on dental health outcomes, Lee and colleagues developed the Rapid Estimate of Adult Literacy in Dentistry (REALD-30), which is a specific instrument for assessing the level of literacy on oral health through an analysis of word recognition[9]. The REALD-30 is made up of 30 common dental words arranged in ascending order of difficulty based on the Rapid Estimate of Adult Literacy in Medicine (REALM), a 66-item word-recognition screening tool that is quick and easy to administer, designed for use in public health and primary care to identify patients with low reading skills [13].

The association between better reading skills and a higher level of literacy seems obvious, since literacy is defined as ability to read and write. However, a low correlation has been found between schooling and literacy. Thus, educational level may not reflect an individual's ability to obtain, process and understand basic health information, which may exert an influence on health outcomes and appropriate decision making [1,8,9]. It is therefore important to assess functional health literacy to determine an individual’s ability to understand basic health information, considering the important role of literacy in health outcomes[1,9].

Data from the last National Oral Health Survey [14] show that despite the important reduction in dental caries index and the better oral indicators observed, the oral health status of the Brazilian population, especially of children (the mean number of decayed, missing, or filled teeth in 5-year-old children was 2.3, with 80% of decayed deciduous teeth still untreated), is still far short of the goals proposed by the World Health Organization, highlighting the need for efforts to improve this situation. Therefore, oral health literacy can be an important tool to enhance the communication skills between population and oral health professionals in this country.

The REALD-30 was developed in an English-speaking country and may be subject to the influence of local culture. For an instrument to be used in other contexts and countries, it must undergo a translation, cross-cultural adaptation and validation process[15]. Thus, the aim of the present study was to translate, perform the cross-cultural adaptation of the REALD-30 to Brazilian-Portuguese language, and test the reliability and validity of this version.

## Methods

The study was approved by the Committee for Ethics in Human Research of Universidade Federal do Parana (Brazil) and carried out according to the Declaration of Helsinki. Written informed consent was obtained from the volunteers prior to data acquisition procedures.

### Description of the REALD-30

The REALD-30 is a specific instrument for assessing the level of literacy of adults regarding oral health through the recognition of words related to the etiology, anatomy, prevention and treatment of adverse oral conditions. The instrument contains 30 words to be read aloud by the respondent to the interviewer. The list of words is arranged in ascending order of difficulty based on both the average word length, number of syllables and the difficulty of combining sounds. For each word pronounced correctly, one point is assigned to the REALD-30 score and zero is recorded when the pronunciation is incorrect. The total score is obtained by summing the scores and ranges from 0 (lowest degree of literacy) to 30 (highest degree of literacy)[9].

### Other measures

In addition to the Brazilian version of the Rapid Estimate of Adult Literacy in Dentistry (BREALD-30), each subject (parents/caregivers of children in treatment at pediatric dental clinics or health units) also participated in an interview that involved the administration of items from the National Functional Literacy Index (NFLI),which assesses levels of general literacy among the adult population in Brazil based on a 20-question test of reading and comprehension skills. NFLI items cover all levels of general literacy and are arranged in ascending order of difficulty. The scores for each item are summed to obtain the total score, with higher scores corresponding to a higher level of general literacy [16].

The participants answered a questionnaire addressing demographic data related to the child [birth date and gender (male, female)] and related to the parent/caregiver [age (years), gender (male, female), educational attainment (number of years of school completed), marital status (single, married, stable relationship, widow, divorced)and occupation (free answer)], socioeconomic status [monthly household income in Brazilian currency (Reais)–categorized later based on the monthly minimum salary in Brazil], use of dental services [“When was the last time you visited a dentist?” (within the past year; within 2 years; within 5 years; greater than 5 years; or never)], oral health perceptions [self-rated oral health and respondent’s perception regarding his/her child's oral health (assessed on a five-point scale: excellent, very good, good, fair, and poor)].

The subjects also answered the short-form of the Oral Health Impact Profile (OHIP-14),which is a 14-question oral health-related quality of life (OHRQoL) assessment tool [17]. Each item is scored using a five-point scale ranging from no impact on OHRQoL (score 0) to maximum impact on OHRQoL (score 4).

### Translation and cross-cultural adaptation of the REALD-30

Based on standard recommendations, the translation and cross-cultural adaptation of the REALD-30 were initially performed by two independent translators (a Brazilian fluent in English and a native English speaker fluent in Portuguese) with experience in the translation of health questionnaires [15]. The assessment of the versions was performed in a ‘double-blind’ manner in relation to both the translator and back translator. The translation panel consisted of researchers, two translators, and three dentists, all fluent in both Portuguese and English. The original and back-translated versions were compared by a committee composed of a group of dental specialists with knowledge regarding health education assessments and fluency in the English language. This committee made comments and offered suggestions so that the back-translated items would come as close as possible to those on the original instrument. The assessments made by the committee were reviewed during a consensus meeting.

For the determination of conceptual equivalence, a committee of experts in oral health and health education assessed the relevance of the items on the Brazilian-Portuguese language version in comparison to the original English language version. The committee evaluated whether the areas covered by the original instrument regarding the concepts of interest would be relevant and pertinent to the cultural context to which the REALD-30 was being adapted.

The BREALD-30 was first pre-tested on a convenience sample of 10 individuals (parents/caregivers of children in treatment at the pediatric dental clinics of the Universidade Federal do Parana) aged 19 to 56 years with different levels of education. Attention was given to the meaning of the words in the different languages to obtain similar effects from respondents of different cultures. A synthesis of the instrument was developed as a result of this process.

The original instrument has two essential characteristics: words related to adverse oral conditions (etiology, anatomy, treatment and prevention) and arranged in ascending order of reading difficulty. However, the literal translation did not ensure the maintenance of the latter feature. Reading difficulty is related to the structure of the word and commonly used words, which are often changed during a translation process due to linguistic or cultural differences. Moreover, some words had a double meaning when translated into Portuguese, allowing their association with both dentistry and other contexts. Thus, the translated instrument demonstrated little power of discrimination and some words of the original instrument needed to be replaced in the BREALD-30. A survey was conducted in the audiovisual media, newspapers and the Brazilian trade to identify new terms associated with oral health. This strategy was similar to that employed in the construction of the original instrument [9]. Words related to oral health were ranked according to the frequency of observation and level of difficulty in pronunciation. Twenty words were selected from this survey and added to the 30 words translated from the REALD-30. The new list of 50 words was applied to a group of 14 individuals (parents/caregivers of children in treatment at the pediatric dental clinics) aged 25 to 67 years (2^nd^ pre-test). Two aspects were considered to determine which words in the original instrument should be replaced: ambiguity and readability.

To maintain the same proportion of easy, moderate and difficult words suggested by the authors of the original instrument [18], the words *fluoride*, *extraction* and *caries* were replaced with more complex words. Moreover, the words *braces* and *floss* were replaced because their translation into Portuguese generated compound words, which are not found on the original instrument. As a result of this process, the words *cellulitis*, *pulp*, *sealant*, *fluoride*, *extraction*, *caries*, *braces* and *floss* were replaced with *bleeding*, *endodontics*, *film*, *mouthwash*, *biopsy*, *erosion*, *orthodontics* and *radiography*.

To assess the transference of meaning between the original and the translated versions, two native English-speaking individuals, who were not previously involved in the study, performed the back translation into English of the synthesis version. Although the final version is not a literal translation of the original instrument, the two back-translated English versions proved that the original goals were maintained. To determine semantic equivalence, three experts in oral health and health education [fluent in both languages (English and Portuguese) and with no prior knowledge of the study] compared the back-translated English version to the original English language version. The aim of this step was to achieve a ‘similar effect’ from respondents who speak English and Portuguese [15].

During the second pre-test, evaluations were made regarding the possibility of maintaining the operational characteristics of the original instrument in the translated version and whether the instructions, mode of administration and measurement methods were similar to the original English version.

### Assessment of the validity and reliability of the BREALD-30

For the assessment of the psychometric properties of the instrument, 258 parents/caregivers of children treated at pediatric dental clinics and health units in the metropolitan region of Curitiba (Brazil) were interviewed from February to September 2012. The individuals also participated in an interview that involved questions from the NFLI, the OHIP-14 and a questionnaire addressing demographic data, socioeconomic status, the use of dental services and oral health perceptions. The interviews were conducted by a single investigator (MCJ) who had previously undergone a training process for the interview protocol.

All subjects enrolled in the validation study of the BREALD-30 were adults aged 18 to 75 years who had Brazilian-Portuguese as their native language. Illiterate individuals and those with cognitive impairment, uncorrected vision or hearing impairment or obvious signs of intoxication by drugs or alcohol at the time of interview were excluded.

**Fig 1:**
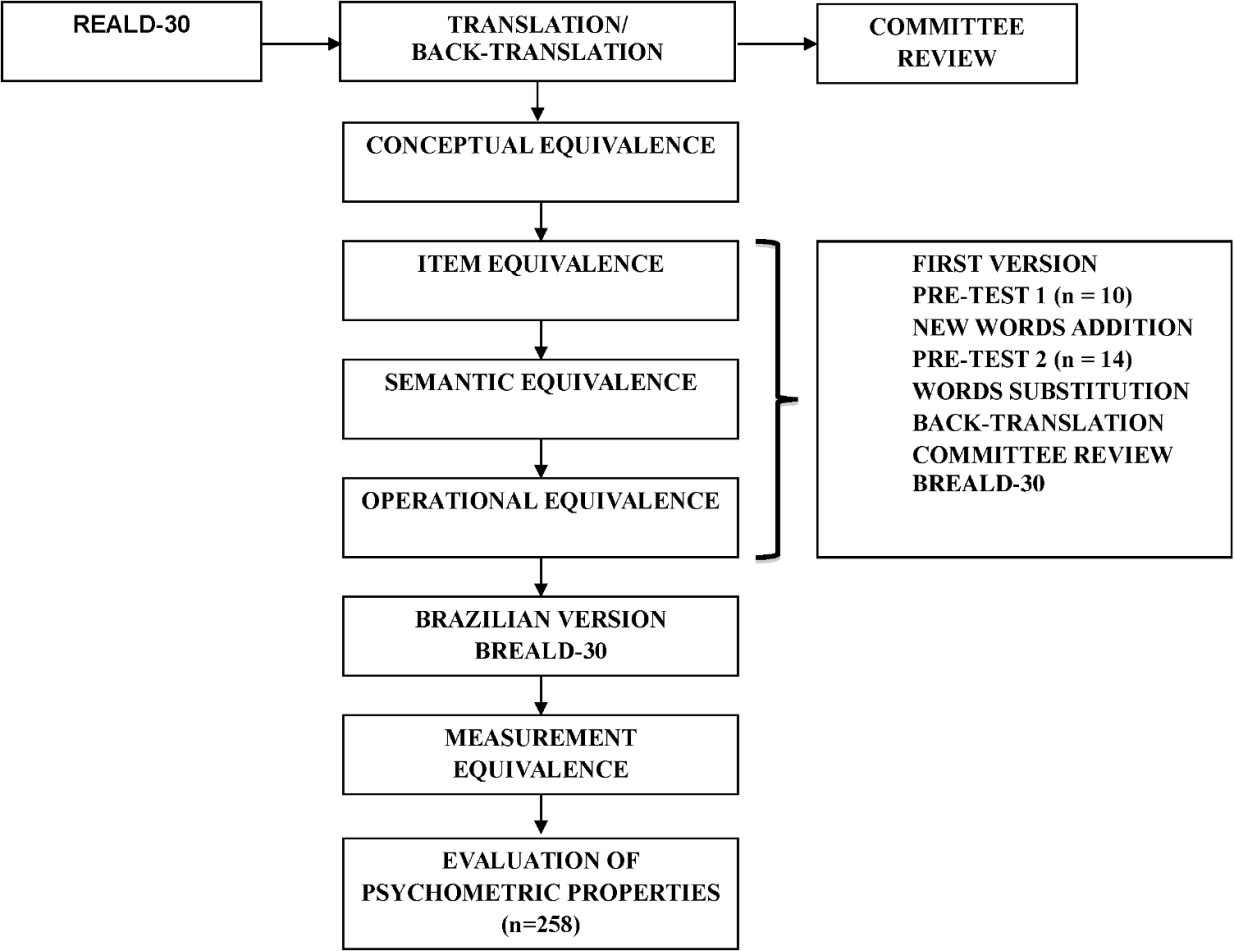
Flow chart of the cross-cultural validation steps.

A synthesis of the translation, cross-cultural adaptation and validation of the REALD-30 process could be visualized on Fig 1.

### Statistical analysis

For the statistical analysis, the participants were separated into categories based on marital status (married/otherwise), gender (male/female), educational attainment [≤ 8 years of study (elementary school)/> 8 years of study], occupation (related/unrelated to health field), last dental visit (in the last year/more than a year ago), self-rated oral health [good (excellent, very good, good or fair)/poor] and the respondent’s assessment on his/her child’s oral health [good (excellent, very good, good or fair)/poor]. Age in years and monthly household income were also considered, the latter of which was based on the Brazilian minimum salary (roughly equal to US $258.33 at time of data collection).

SPSS Statistics (version 20.0, SPSS Inc., Chicago, Illinois, USA) was used for data analysis. Descriptive analyses were performed (mean, median, standard deviation and total BREALD-30 score for each participant). Internal consistency of the BREALD-30 (total and inter-item) was tested using Cronbach’s alpha coefficient [19]. Test-retest reliability was assessed on a word-by-word basis by calculating the Kappa coefficient and for the BREALD-30 score using the intraclass correlation coefficient (ICC), with data from the reapplication of the instrument to 10% of the sample one month after the first interview.

The assumption of unidimensionality of the instrument was tested by exploratory factor analysis [20]. Oral health literacy was considered one-dimensional, thereby representing one factor. Eigenvalues, which represent the variance in a set of variables described by a factor (in the present case, dental health literacy) ​​generated from the factor analysis were considered to determine whether there was a dominant factor among the words on the BREALD-30. Eigenvalues greater than one were considered a factor.

Convergent validity was accessed by correlating the BREALD-30 scores with the NFLI and educational attainment. Discriminant validity was tested by comparing BREALD-30 scores according to the categorized groups for occupation and use of dental services, as well as monthly household income. As BREALD-30 scores were not normally distributed, the non-parametric Wilcoxon test, Mann-Whitney test and Spearman’s correlation coefficient were used in these analyses.

To determine predictive validity, the hypothesis was that the BREALD-30 score was associated with dental outcome measures (oral health status: self-rated oral health and respondent’s assessment of his/her child’s oral health) and the impact of oral conditions on quality of life measured by the OHIP-14. The influence of BREALD-30 on these oral health-related variables could be explained by the fact that, according to the model developed by Guo et al. [21], higher health literacy levels were associated with better patient-dentist communication, which in turn corresponded with being a regular (rather than problem-oriented) dental care seeker, and finally being these better dental care pattern associated with better self-rated oral health. To test this hypothesis, three multiple Poisson regression models with robust variance were performed using the OHIP-14 score and oral health status as the dependent variables and the BREALD-30 score as the major independent variable. Gender, age, educational attainment, marital status and history of dental visits were incorporated into the models for adjustment purposes, since the literature states that these variables can be associated with the outcomes [9]. The model for the respondent’s assessment of his/her child’s oral health was also adjusted for child’s gender and age. The level of significance was set at 5%.

## Results

The validity and reliability assessments of the BREALD-30 were conducted with 258 parents/caregivers aged 18 to 75 years [mean: 33.8, Standard Deviation (SD): 11.7 years; 23 males (9%) and 235 females (91%)]. The age of the children for whom the respondents were responsible ranged from one month to 12 years (mean: 6, SD: 3.7 years). The mean BREALD-30 score was 21.6 (SD: 5.5). The mean NFLI score was 7.3 (SD: 1.9) and the mean OHIP-14 score was 16.0 (SD: 11.9).

The BREALD-30 demonstrated good internal reliability. Cronbach's alpha ranged from 0.88 to 0.89 when words were deleted individually. The analysis of test-retest reliability demonstrated excellent reproducibility [ICC = 0.983 (95% CI: 0.963 to 0.993) and Kappa coefficients ranging from moderate to nearly perfect (0.42–1.00)].

Regarding construct validity, the exploratory factor analysis of the words on the BREALD-30 demonstrated the undeniable predominance of one factor. The eigenvalue for the first factor (7.36) was approximately fourfold greater than that for the second factor (1.88), which was similar to the eigenvalue for the third factor (1.62). The scree plot also demonstrated the clear predominance of one factor. Based on the Kaiser criterion, only factors with eigenvalues greater than one were extracted; as those with lower values contribute little to explaining the variance in the original variables [22]. Factor I (nine words) accounted for 24.5% of the variance and included the words *analgesia*, *endodontics*, *malocclusion*, *abscess*, *fistula*, *hyperemia*, *orthodontics*, *hypoplasia* and *apicectomy*. Factor II accounted for 6.3% of the variance and included the words *teeth*, *erosion*, *restoration*, *biopsy*, *bruxism*, *periodontal* and *film*. A minimum of seven factors were necessary to explain 50% of the total variance.

The BREALD-30 demonstrated satisfactory convergent validity, as the scores correlated with the level of general literacy measured by the NFLI (*r*_*s*_ = 0.593; p < 0.001) and educational attainment (*r*_*s*_ = 0.541; p < 0.001).

Discriminant validity was determined by comparing BREALD-30 scores according to occupation (Mann Whitney, p = 0.004), a history of dental visits (Mann Whitney, p = 0.017) and monthly household income (*r*_*s*_ = 0.327 and p < 0.001), with statistically significant differences in the scores among the different groups.

Regarding predictive validity, no correlation was found between the BREALD-30 and OHIP-14 scores (*r*_*s*_ = -0.080; p = 0.198), but the BREALD-30 score was statistically associated with self-rated oral health and respondent’s assessment of his/her child’s oral health in the bivariate analysis (Mann Whitney, p = 0.003 for both variables). However, after adjusting for other covariates in the multiple Poisson regression, only the association between the BREALD-30 score and the respondent’s assessment of his/her child’s oral health remained significant (Table 1).

**Table 1:** Multiple Poisson regression to assess the association between BREALD-30 and dental outcomes (n = 258).

Measures	OHRQoL (OHIP-14)		Negative self-rated oral health		Parent’s/guardian’s negative perception of child's oral health	
	p-value	Adjusted RR (95% CI)	p-value	Adjusted PR (95% CI)	p-value	Adjusted PR (95% CI)
BREALD-30 score	0.064	0.98(0.97–1.00)	0.083	0.97(0.94–1.00)	**0.024**	**0.94(0.88–0.99)**
*Control variables*						
Gender (male* / female)	0.376	0.84(0.58–1.23)	0.973	1.01(0.53–1.95)	0.435	0.47(0.07–3.17)
Age (in years)	**0.002**	**1.01(1.01–1.02)**	0.793	0.99(0.98–1.02)	0.118	0.95(0.90–1.01)
Marital status (married*/otherwise)	0.707	1.04(0.84–1.30)	0.252	0.76(0.47–1.22)	0.198	0.58(0.25–1.34)
Educational attainment (>8 years*/≤8 years)	0.216	0.89(0.73–1.07)	0.065	0.64(0.39–1.03)	0.112	0.50(0.21–1.18)
Dental visit (in the last year* / more than 1 year ago)	0.383	1.08(0.90–1.30)	**0.031**	**0.64(0.43–0.96)**	0.316	0.66(0.30–1.48)
Child’s gender (male* / female)	-	-	-	-	0.613	1.23(0.55–2.75)
Child’s age (in years)	-	-	-	-	**0.004**	**1.22(1.07–1.40)**

*Reference category

Significant results at 5% level in bold type; OHRQoL: oral health-related quality of life; RR: rate ratio; PR: prevalence ratio; CI: confidence interval (obtained by adjusted Poisson regression with robust variance)

## Discussion

The psychometric properties of the BREALD-30 were similar to those of the original instrument [9]. The BREALD-30 demonstrated satisfactory validity and reliability, indicating its adequacy for use on the Brazilian population. The administration of the instrument is relatively simple and quick (up to three minutes), requires minimal training and can be administered in clinical settings.

The BREALD-30 demonstrated excellent internal consistency, as Cronbach's alpha coefficient (0.88) was similar to that of the original instrument, the validation of the Hong Kong (HKREALD-30) and ArabicREALD-30 (AREALD-30) (0.87; 0.84 and 0.89 respectively) [9,23,24]. A Cronbach’s alpha coefficient ranging from 0.5 to 0.7 is generally considered satisfactory for comparisons between groups and values greater than 0.85 are sufficiently reliable for comparisons on the individual level [25].

Test-retest reliability was assessed using the ICC. The results demonstrated the excellent stability of the instrument (ICC = 0.98; Kappa coefficient = 0.42 to 1.00), whereas the ICC for the HKREALD-30 and AREALD-30 were 0.78 and 0.99 [23,24].

Similar to the original instrument, the BREALD-30 demonstrated adequate convergent validity. The scores were significantly and positively correlated with the scores of the assessment tool used to measure general literacy (NFLI) and educational attainment. In the original REALD-30, two different instruments were used to assess health literacy: the REALM and TOFHLA [9]. These instruments were not employed in the present study because they have not been validated for Brazilian Portuguese. The association between a low degree of dental health literacy and low educational attainment has also been reported in the literature [9,26,27].

As the cutoffs for establishing levels of dental health literacy were not defined by the authors of the original instrument (REALD-30), the results of the BREALD-30 were treated as a continuous scale with a mean score of 21.6 (SD: 5.5). Studies available in the literature that have applied the REALD-30 report mean scores ranging from 15.8 (SD: 5.3) to 23.9 (SD: 1.3) [5,27].

Regarding construct validity, although the exploratory factor analysis demonstrated the clear predominance of one factor (eigenvalue around fourfold greater than that of the second factor), the hypothesis that dental health literacy measured by the words of the BREALD-30 is unidimensional was not confirmed. It took at least seven factors to explain 50% of the total variance. These results are similar to the original study, which also identified the predominance of one factor, the eigenvalue of which was more than fourfold greater than that of the second factor, with the presence of at least one more factor. The authors of the REALD-30 suggest some possible explanations for the multidimensionality of dental health literacy. It is believed that different domains are related to differences in reading ability and the difficulty of the words[9]. This explanation seems consistent when observing the words included in factor I (*analgesia*, *endodontics*, *malocclusion*, *abscess*, *fistula*, *hyperemia*, *orthodontics*, *hypoplasia* and *apicectomy*), all of which were in the group of greatest difficulty. The hypothesis seems adequate if one considers that the presence of domains related to different levels of difficulty can increase the discriminating power of a test that assesses the ability to recognize words. However, further studies are needed to confirm and understand this hypothesis.

Individuals with a health-related occupation, those who visited the dentist in the previous year and those with a higher household income had higher degrees of dental health literacy, which suggests that the BREALD-30 may be capable of discriminating subjects. Previous studies also found that not having visited a dentist in the previous year [26] and having a low socioeconomic status [26,28]were associated with a low degree of dental health literacy. Discriminant validity was not evaluated in the original study [9].

As in the original instrument, the BREALD-30 was associated with at least one oral health outcome, which suggests predictive validity [9]. Associations were found between the BREALD-30 score and both self-rated oral health (bivariate analysis) and respondent’s assessment of his/her child’s oral health (bivariate and multivariate analysis). Some studies have shown an association between a low degree of dental health literacy and negative self-rated oral health [5,23,26], others have found low caregiver literacy to be linked to poorer child oral health outcomes and more detrimental oral-health-related behaviors, including worse caregiver's reports of their child's oral health status [29,30]. Word recognition and reading comprehension may affect the understanding regarding instructions and options involving health, which can compromise health-related decision making [9,28].

The subjects included in the present study were recruited from environmental health services. During data collection, most children were accompanied on treatment by women, which is very characteristic of Brazilian society. As a result, the sample consisted almost of females (only 23 men were interviewed). This is a limitation of the study, due to the fact that a sample recruited in a clinical setting may have greater access to healthcare information than the general population and, mainly, there could be differences in male and female literacy levels. Even though there were no consensus on the literature about differences for gender regarding health literacy [31,32,33], it may play a role not only on literacy, but also on the other variables considered in this study. So it is not possible to assume that the validity of BREALD-30 operates equivalently across gender. Further investigations enrolling subjects from both genders and different segments of society are required, as nonrandom sampling has a limited ability to represent the population.

Moreover, the BREALD-30 is an instrument that only assesses one dimension of dental health literacy–reading ability or word recognition. There are basically three main types of instruments developed for oral health literacy assessment: word recognition instruments; reading comprehension and numeracy; and conceptual knowledge, the former being most common [34]. In this type of instrument, the words are displayed out of context, without assessing reading comprehension. This is a characteristic also of the original REALM and REALD instruments. Recently, some studies and a systematic review in this area have pointed out the need to invest in the development of valid and reliable tools to evaluate the functional and conceptual literacy in oral health, which allow that all patients' levels of literacy in oral health be more accurately evaluated in clinical practice [21,34,35,36].

The Brazilian version of the REALD-30 demonstrated satisfactory psychometric properties and proved to be a rapid, simple and reliable measure of dental health literacy among adults who speak Brazilian Portuguese. The BREALD-30 may be used for screening on an individual level to identify patients with low degrees of dental health literacy, thereby allowing dental health professionals to adjust their communication strategies for each patient. The instrument can be also used in association with other indicators to better assess the dental health literacy on community level, providing information to health administrators that can allow the development of more appropriate educational approaches.
